# Renin Angiotensin Aldosterone System Blockades Does Not Protect Residual Renal Function in Patients with Hemodialysis at 1 Year After Dialysis Initiation: A Prospective Observational Cohort Study

**DOI:** 10.1038/s41598-019-54572-6

**Published:** 2019-12-02

**Authors:** Kyung Don Yoo, Clara Tammy Kim, Soie Kwon, Jeonghwan Lee, Yun Kyu Oh, Shin-Wook Kang, Chul Woo Yang, Yong-Lim Kim, Yon Su Kim, Chun Soo Lim, Jung Pyo Lee

**Affiliations:** 1 Department of Internal Medicine, Ulsan University Hospital, Ulsan, Korea; 2 Institute of Life and Death Studies, Hallym University, Chuncheon, Korea; 3 Department of Internal Medicine, Seoul National University Hospital, Seoul, Korea; 4 Department of Internal Medicine, Seoul National University Boramae Medical Center, Seoul, Korea; 5 Department of Internal Medicine, Seoul National University College of Medicine, Seoul, Korea; 6 Department of Internal Medicine, Yonsei University College of Medicine, Seoul, Korea; 7 Department of Internal Medicine, The Catholic University of Korea College of Medicine, Seoul, Korea; 8 Department of Internal Medicine, Kyungpook National University School of Medicine, Daegu, Korea

**Keywords:** End-stage renal disease, Haemodialysis

## Abstract

The beneficial effects of renin angiotensin aldosterone system (RAAS) blockade on residual renal function (RRF) in patients who have just initiated hemodialysis (HD) have been inconclusive. In this study, 935 patients with incident HD from a nationwide prospective observational cohort in Korea were included for analysis. The primary outcome showed that RRF as demonstrated by urine volume changes over 0, 3, and 12 months differed between the RAAS blockade and control groups. Mixed-effects linear regression was used to compare RRF between the groups. Patients in the RAAS group had a greater proportion of higher urine volume at study enrollment compared to the control group, but there was no difference in baseline characteristics, heart function, and dialysis-related indices. After adjusting for confounding factors, the RAAS group did not provide a significant benefit to RRF in a mixed-effects linear regression (p = 0.51). Male gender, high Charlson comorbidity index, diuretic use, and high weekly ultrafiltration volume were associated with faster decline in RRF. The RAAS group failed to provide a protective effect for the development of anuria 1 year after initiating dialysis based on the multivariate logistic regression (OR 0.73 95% CI 0.25–2.13, p = 0.57). In Korean patients with incident HD, RAAS blockade did not provide a protective effect for RRF after 1 year. Further research is needed to clarify the optimal treatment for preserving RRF in HD patients.

## Introduction

The residual renal function (RRF) of a dialysis patient gradually decreases and disappears as dialysis duration increases. RRF may have many benefits for patients with end-stage renal disease (ESRD) on hemodialysis (HD), including associations with better mortality and morbidities, not only in patients who have just initiated HD^1,2^, but also in those on maintenance HD^3^. Preserved kidney function offers HD patients several advantages, such as a lower dialysis dose, more liberal diet intake, and a lower ultrafiltration rate per dialysis session^4–6^. Despite these benefits, there are few data on HD patients compared to peritoneal dialysis patients^7^, so the importance of RRF has increased in HD patients^8^.

The renin angiotensin aldosterone system (RAAS) blockade is the first-line choice for blood pressure control and for delaying the progression of chronic kidney disease (CKD) according to the KDIGO^9^ and KDOQI^10^ guidelines. However, controversies remain, especially in advanced CKD for the overlooked risk of using an RAAS blockade^11^, and whether RRF is spared in HD patients. Previous studies of RAAS blockade in patients on HD focused mainly on cardiovascular organ damage or mortality and did not consider RRF^12^. An observational study of newly started HD patients from the US Renal Data System database found a protective effect of RAAS blockade on the decline of RRF, as defined by urine volume, by Moist *et al*.^13^. The results of subsequent studies are inconsistent^13–17^. Although a small observational study by Itoh *et al*.^14^ showed that RAAS blockade protects RRF, a randomized controlled trial (RCT) using the angiotensin-receptor blocker (ARB) irbesartan failed to show a protective effect compared to placebo^15^. Although dialysis-related indices—such as Kt/v—were not analyzed or included in patients receiving maintenance HD in that study, there were no differences between the groups^15^.

The association between RAAS blockade and its benefit or harm to RRF in HD patients has been evaluated, but much of the existing literature is limited by small sample size^14–16^, retrospective study design with random sampling^13^, or a lack of a standardized definition and measurements of RRF^4^. Moreover, the concurrent medications for RRF, such as diuretics, have not been well evaluated despite their extensive use. In this study, we primarily assessed the clinical advantages of an RAAS blockade in treating hypertension in RRF among patients who have just initiated HD.

## Results

### Between-group differences in baseline characteristics between the RAAS and control groups

**Figure 1:**
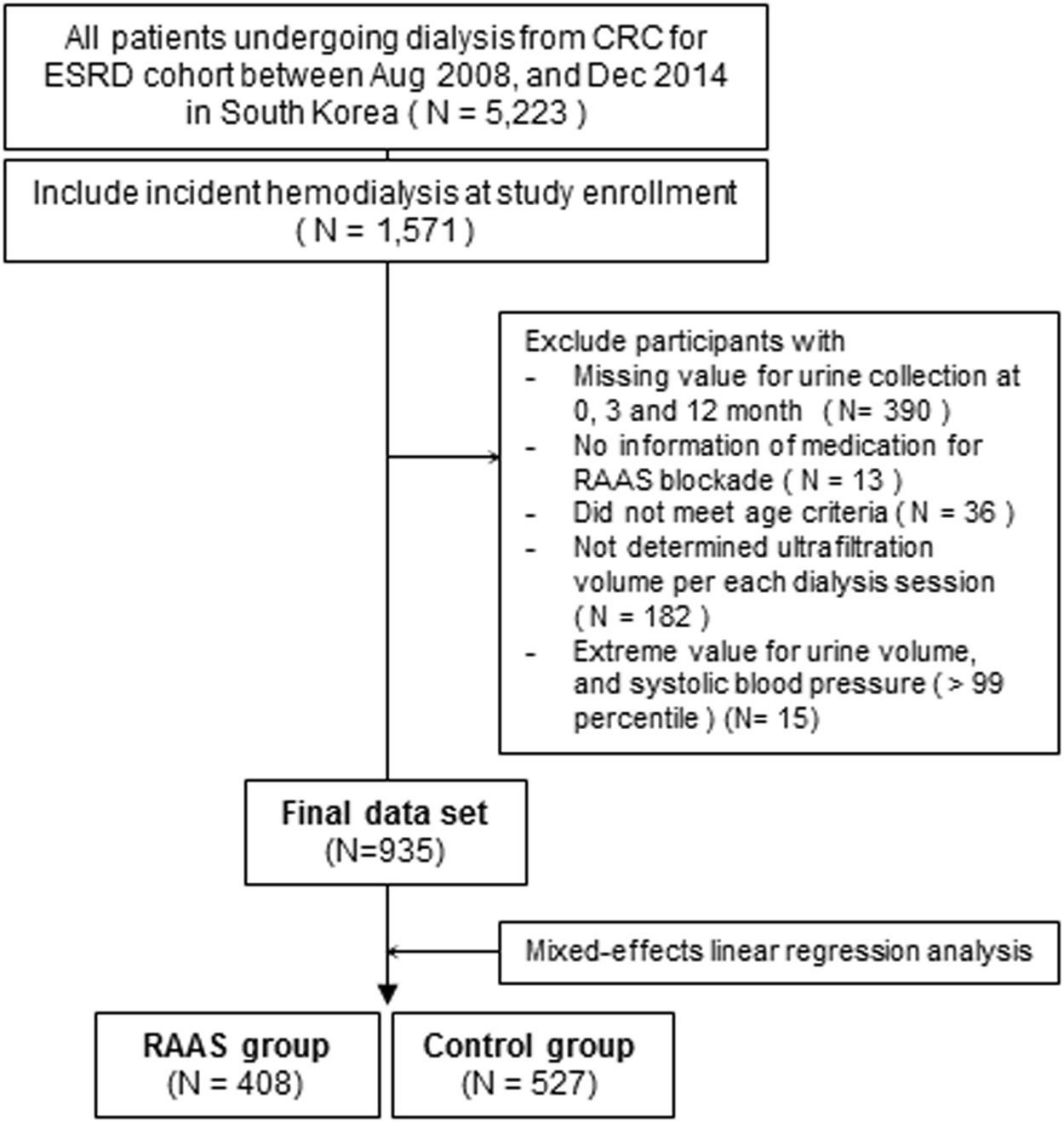
Study flow chart CRC for ESRD cohort, Clinical Research Center for End-Stage Renal Disease cohort; RAAS blockade, Renin-Angiotensin-Aldosterone System blockade.

Demographic and baseline clinical characteristics were assessed and are presented in Table 1. Of all patients, 60.1% were male and mean age was 57.6 ± 13.9 years old. Among patients with incident HD, 408 were classified to the RAAS group and 527 were classified to the control group (Fig. 1). Between-group differences in age, sex, primary renal diseases, and comorbidities were not significant and are summarized as follows: Patients in the RAAS group were 57.62 ± 14.04 years old, whereas subjects in the control group were 57.60 ± 13.95 years old (p = 0.98), and had a higher likelihood of diabetes from primary renal disease of ESRD (54.9% in the RAAS group vs. 50.3% in the control group; p = 0.57). No significant between-group differences in smoking, cardiovascular disease history, or blood pressure were observed. Patients in the control group used a greater number of diuretics for volume control (49.8% in the RAAS group vs. 57.1% in the control group; p = 0.02), and on the contrary, patients in the RAAS group used a greater number of calcium channel blockers (66.7% in the RAAS group vs. 60.9% in the control group, respectively; p = 0.07). A similar proportion of patients in the control group used beta and alpha blockers for blood pressure control compared to the RAAS group (Table 1).

**Table 1:** Baseline characteristics based on RAAS blockade usage.

Variables	Total N = 935		
	Control group (N = 527) (%)	RAAS group (N = 408) (%)	*P*
Age (years old)	57.60 ± 13.95	57.62 ± 14.02	0.98
Sex (male)	312 (59.2)	250 (61.3)	0.52
Primary renal disease			0.57
Diabetes	265 (50.3)	224 (54.9)	
Hypertension	65 (12.3)	54 (13.2)	
Glomerulonephritis	73 (13.9)	53 (13.0)	
Cystic kidney disease	15 (2.8)	9 (2.2)	
Unknown	39 (7.4)	27 (6.6)	
Others	70 (13.3)	41 (10.0)	
History of CVD	152 (28.8)	120 (29.4)	0.84
History of DM	299 (56.7)	241 (59.1)	0.47
Current smoking history (%)	47 (8.9)	49 (12.0)	0.12
SBP (mmHg)	142 ± 21	144 ± 22	0.12
DBP (mmHg)	77 ± 14	78 ± 14	0.42
BMI (kg/m2)	23.01 ± 3.64	23.22 ± 3.46	0.36
Modified CCI	5.45 ± 2.24	5.35 ± 2.41	0.50
**Concurrent-antihypertensive medications (%)**			
Calcium channel	321 (60.9)	272 (66.7)	0.07
B-blockers	279 (52.9)	229 (56.1)	0.33
Diuretics	301 (57.1)	203 (49.8)	0.02
a-Blockers	65 (12.3)	54 (13.2)	0.68
**Cardiologic evaluation**			
LVH on ECG	133 (25.2)	99 (24.3)	0.73
cTnT	0.61 ± 7.09	1.24 ± 11.47	0.43
NT pro-BNP (pg/mL)	17,470 ± 33,856	16,409 ± 22,218	0.69
**Echocardiographic parameter**			
LAD (cm)	4.22 ± 0.77	4.30 ± 0.68	0.20
LVESD (cm)	3.51 ± 0.82	3.53 ± 0.72	0.72
LVEDD (cm)	5.06 ± 0.84	5.16 ± 0.70	0.09
LVMI (g/m^2^)	229.58 ± 376.43	250.58 ± 412.00	0.52
Ejection fraction (%)	57.29 ± 12.17	59.53 ± 10.45	0.01

CVD, cardiovascular disease; DM, diabetes mellitus; SBP, systolic blood pressure; DBP, diastolic blood pressure; MCCI, modified Charlson comorbidity index; LVH, left ventricular hypertrophy; cTnT, cardiac troponin T; NT pro-BNP, N-terminal pro-B-type natriuretic peptide; RAAS blockade, renin-angiotensin-aldosterone system blockade; LAD, left atrial dimension; LVESD, left ventricular end-systolic dimension; LVEDD, left ventricular end-diastolic dimension; LVMI, left ventricular mass index.

*Values are presented as n (%) for categorical variables, mean ± standard deviation for continuous variables.

In this study, the enrolled patients underwent a cardiology examination including 2D echocardiography and ECG. The left ventricular end-diastolic dimension (LVEDD) on 2D echocardiography was increased in the control group compared to the RAAS group (5.16 ± 0.70 cm in the RAAS group vs. 5.06 ± 0.84 cm in the control group; P = 0.09). All other cardiac parameters were comparable between the RAAS and control groups. Notably, the mean B-type natriuretic peptide level was high in both groups, with levels of 17,470 ± 33,856 pg/mL for the control group and 16,409 ± 22,218 pg/mL for the RAAS group (Table 1).

### Between-group differences in dialysis-related indices and RRF between the RAAS and control groups

The results are shown in Table 2. No significant differences in single-pool Kt/V, weekly Kt/V, urea reduction rate (URR), or ultrafiltration volume (UF) per session and week were observed between the groups. The single-pool Kt/V was 1.36 ± 0.53 in the RAAS group and 1.36 ± 0.44 in control group, and the UF volume per session was 1.50 ± 1.39 in the RAAS group and 1.50 ± 1.39 in the control group. Both single pool Kt/V and URR exceeded the recommendations of the KDOQI^18^.

**Table 2:** The comparison of dialysis-related indices and baseline residual renal function (RRF) between the RAAS group and control groups.

Variables	Total N = 935		
	Control group (N = 527)	RAAS group (N = 408)	*P*
**Dialysis-related indices**			
Single-pool Kt/V	1.36 ± 0.44	1.36 ± 0.53	0.95
Weekly Kt/V	3.83 ± 1.60	3.85 ± 1.66	0.87
Urea reduction rate (%)	67.10 ± 9.44	66.45 ± 10.06	0.43
UF/session (kg)	1.50 ± 1.30	1.50 ± 1.39	0.94
Weekly UF (kg)	4.37 ± 3.90	4.32 ± 4.06	0.84
**Intradialysis-related indices**			
Pre-dialysis SBP	143 ± 22	145 ± 23	0.16
Pre-dialysis DBP	75 ± 13	76 ± 14	0.40
Post-dialysis SBP	144 ± 22	149 ± 21	<0.001
Post-dialysis DBP	76 ± 13	78 ± 13	0.01
Minimum SBP during dialysis	134 ± 23	139 ± 53	0.03
Minimum DBP during dialysis	73 ± 13	74 ± 14	0.55
Intradialytic hypotension (%)^*^	188 (35.7)	141 (34.6)	0.72
Urine volume per 24 h at the time of study enrollment (0 month)			0.04
More than 500 ml	249 (47.2)	231 (56.6)	
<500 ml	84 (15.9)	52 (12.7)	
<100 ml	77 (14.6)	52 (12.7)	
No data	117 (22.2)	73 (17.9)	

UF, ultrafiltration; SBP, systolic blood pressure; DBP, diastolic blood pressure; RAAS blockade, Renin-angiotensin-aldosterone system blockade.

^*^Intradialytic hypotension, A decrease in systolic BP ≥ 20 mm Hg or a decrease in mean arterial pressure (MAP) ≥ 10 mm Hg, compared to predialysis BP during dialysis.

^¶^Anuria, 24-h urine volume < 100 ml.

Blood pressure changes during HD were also recorded. Both systolic and diastolic blood pressures measured before and after dialysis were higher in the RAAS group compared to the control group. In particular, both systolic blood pressure after dialysis (post-dialysis SBP) (144 ± 22 mmHg in control group vs.149 ± 21 mmHg in RAAS group, p < 0.001) and minimum systolic blood pressure (139 ± 53 mmHg in control group vs.134 ± 23 mmHg in RAAS group, p = 0.003) were higher in the RAAS group compared to the control group. There was no other difference in dialysis-related indices during dialysis between the two groups. There was no difference in the frequency of intradialytic hypotension during dialysis (p = 0.72).

Notably, the proportion of patients with preserved urine volume (more than 500 mL per 24 h) was higher in the RAAS group than in the control group (56.6% in the RAAS group vs. 47.2% in the control group, p = 0.04) (Table 2).

### RRF change over 0, 3, and 12 months between the RAAS and control groups

Patients were classified into the RAAS group if they used an angiotensin-converting enzyme inhibitor (ACEi) or angiotensin-receptor blocker (ARB) at enrolment and continued the medications for at least 3 months. We performed additional analyses on RAAS blockade use to thoroughly assess the RAAS effect on RRF using 12-month urine volume data. RRF was defined as 24-h urine volume at 0, 3, and 12 months after initiating dialysis.

**Figure 2:**
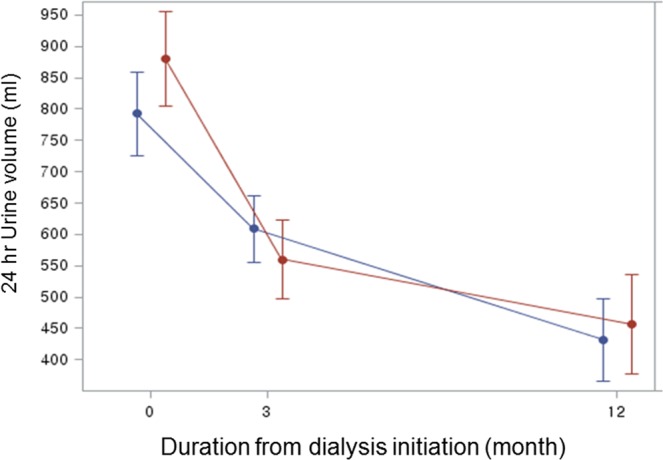
Mixed-effects linear regression analysis between the RAAS group and control group and the association with residual renal function 1 year after initiating dialysis. The adjusted variables included age, sex, dialysis duration, diuretic use, ultrafiltration volume per week, systolic BP, and the modified Charlson comorbidity index. Red line: RAAS group, Blue line: control group.

Least-square means for estimates of urine volume and 95% confidence intervals (CIs) at 0, 3, and 12 months are presented using generalized linear mixed models analysis (Fig. 2). During the study period, a decrease in urine volume across time was observed in all groups. Mean urine volume at baseline (0 month) was 880.39 mL (95% CI 804.96–955.83), and this decreased by 49% to 455.9 mL (95% CI 376.63–535.17) 1 year after dialysis was initiated in the RAAS group (p < 0.001). Mean urine volume at baseline (0 month) was 792.25 mL (95% CI 724.95–859.56), which decreased by 46% to 431.56 mL (95% CI 365.21–497.92) 1 year after initiating dialysis in the control group (p < 0.001). The inter-group difference was not significant (p = 0.51).

Mixed-effects linear regression modeling (LMM) was conducted to investigate variables associated with RAAS blockade and RRF 1 year after initiating dialysis (Table 3). The models were adjusted for age, sex, dialysis duration, diuretic use, UF per week, systolic blood pressure, and the modified Charlson comorbidity index (MCCI). We applied the LMM to two models. Model 1 included patients enrolled in the full analysis (N = 935), and model 2 included only those with urine volume more than 500 ml (N = 480) at the time of the study enrollment. In model 1, after adjusting for covariates, female gender, not use diuretics group, lower weekly UF, and lower MCCI were associated with an lower decrement in urine volume as RRF, whereas the RAAS group did not show the association with preserved RRF (p = 0.51). The interaction between the two fixed effects (RAAS group*Time effect) was significant (p = 0.04) (Table 3). In model 2, after adjusting for covariates, the RAAS group also failed to present the association with preserved RRF (p = 0.15). In addition to applying the LMM model, a sensitivity analysis was applied using the following the definition for the duration of RAAS usage. These results are presented in online Supplement Material (Figs. S1–3).

**Table 3:** The change of urine volume estimates using a linear mixed effect model.

Variables	Model 1^*^	P	Model 2^¶^	P
	Coefficient estimate		Coefficient estimate	
Control group (vs. RAAS group)		0.51		0.15
	−24.33 ± 52.52		42.12 ± 73.38	
Time effect (per visit)		<0.001		<0.001
Visit 0 month (vs. Visit 12)	424.49 ± 54.15		769.68 ± 64.85	
Visit 3 month (vs. Visit 12)	103.99 ± 43.80		197.4 ± 59.33	
RAAS group* Time effect		0.04		0.24
Age (per 1 yr increased)	2.53 ± 1.48	0.08	−1.29 ± 1.67	0.43
Male (vs. female)	−91.14 ± 32.93	0.005	−15.04 ± 38.14	0.69
Not use diuretics group (vs. diuretics use group)	66.11 ± 33.03	0.04	−1.87 ± 37.88	0.96
Weekly UF (per 1 kg increased)	−10.04 ± 4.25	0.01	−4.53 ± 4.77	0.34
SBP (per 1 mmHg increased)	−0.64 ± 0.71	0.37	−0.94 ± 0.83	0.26
Modified Charlson comorbidity index (per 1 increased)	−21.95 ± 8.97	0.01	−3.67 ± 10.65	0.73

UF, ultrafiltration; SBP, systolic blood pressure; DBP, diastolic blood pressure; RAAS blockade, renin-angiotensin-aldost erone system blockade.

Adjusted variable including age, sex, dialysis duration, diuretic use, ultrafiltration volume per week, systolic BP, modified Charlson comorbidity index.

^*^Model 1 presented to the model enrolled in the final analysis using entire study patients (N = 935).

^¶^Model 2 presented to a model in which only the urine volume more than 500 ml was included in the analysis at the time of the study enrollment (N = 480).

### Risk factor analysis for the development of anuria 1 year after initiating dialysis

This risk factor analysis was performed only in the 480 participants with baseline urine volume of 500 ml or more. Their baseline characteristics are presented in the online Supplement Material (Table S1). Over the 1-year follow-up period (12 months from initiating dialysis), the crude rate for anuria was 8.7% in the RAAS group (20 of 231 patients) and 11.2% in the control group among overall participants (28 of 249 patients) (p = 0.345).

Univariate logistic regression analysis of risk factors for anuria development revealed that higher Kt/V (odds ratio 0.29 per 1 increased, 95% confidence interval 0.10–0.83; p = 0.02), higher ejection fraction showed protective effect for RRF (odds ratio 0.15, 95% confidence interval 0.09–0.62; p = 0.005). High ultrafiltration volume per HD session (odds ratio 1.15, 95% confidence interval 0.93–1.43; p = 0.17) showed the possibility of detrimental effect for anuria development. There were no significant effects of intradialytic hypotension (IDH) for RRF (odds ratio 1.35, 95% confidence interval 0.73–2.49; p = 0.33) (Table 4).

**Table 4:** Risk factor analysis for development of anuria after 1year of dialysis initiation using logistic regression analysis

Variables	Univariate	P	Multivariate^*^	P
RAAS group (vs. Control group)	0.74 (0.40–1.36)	0.34	0.73 (0.25–2.13)	0.57
Age	0.98 (0.96–1.00)	0.06	0.99 (0.94–1.05)	0.86
Male (vs. female)	1.35 (0.70–2.61)	0.35	1.20 (0.29–4.89)	0.79
History of CVD	0.95 (0.50–1.82)	0.89	1.18 (0.32–4.33)	0.79
History of DM	0.82 (0.45–1.50)	0.53	0.53 (0.12–2.19)	0.38
Modified CCI	0.94 (0.82–1.07)	0.38	1.03 (0.73–1.47)	0.83
Albumin (g/dl)	0.83 (0.48–1.42)	0.50	0.78 (0.35–1.76)	0.56
**Concurrent-antihypertensive medications (%)**				
Diuretics	1.09 (0.60–2.00)	0.76	1.12 (0.34–3.66)	0.84
**Dialysis-related indices**				
Kt/V (per 1 increased)	0.29 (0.10–0.83)	0.02	0.21 (0.001–63.73)	0.59
Urea reduction rate (per 1 increased)	0.96 (0.93–0.99)	0.01	0.96 (0.83–1.11)	0.66
UF/session (per 1 kg increased)	1.15 (0.93–1.43)	0.17	0.72 (0.42–1.23)	0.24
IDH event (vs. no event)	1.35 (0.73–2.49)	0.33	1.31 (0.43–3.96)	0.62
**Ejection fraction on echoCG**				
Highest quartile (vs. Lowest quartile)	0.15 (0.09–0.62)	0.005	0.44 (0.07–2.75)	0.38

CVD, cardiovascular disease; DM, diabetes mellitus; MCCI, modified Charlson comorbidity index; IDH, intradialytic hypotension; echoCG, echocardiography; RAAS blockade, Renin-angiotensin-aldosterone system blockade.

^*^Participants who urine volume was more than 500 ml for 24 h urine study were included from the final analysis (N = 480).

A multivariate logistic regression analysis revealed that the RAAS group failed to provide an independent association with the protective effect of developing anuria after adjusting for confounders, including age, sex, history of diabetes, cardiovascular disease, MCCI, serum albumin, diuretic use, single-pool Kt/V, URR, UF volume per session, and ejection fraction on 2D-echocardiography (Table 4). Better heart function with higher ejection fraction at dialysis initiation presented protective effect for RRF, but not statistically significantly (odds ratio 0.44, 95% confidence interval 0.07–2.75; p = 0.38) (Table 4).

## Discussion

In this study, we mainly used a linear mixed model analysis and controlled covariates to evaluate the clinical effects for preserving RRF during RAAS blockade treatment among Korean patients undergoing incident HD. The groups were comparable in baseline characteristics, including demographics, concurrent antihypertensive medication use, and 2D-echocardiographic measurements at the initiation of dialysis (Table 1). Interestingly, the RAAS group had a better RRF using 24-h urine volume, compared to the control group (Table 2). The decline in the urine volume estimates 1 year after initiating dialysis using a linear mixed effect model was associated with male gender, a higher UF rate, use of a diuretic, and more comorbid disease (MCCI) (Table 3). The RAAS group failed to provide a protective effect for RRF, compared to the control group (P = 0.51) (Fig. 2).

RRF diminishes over time and eventually disappears in dialysis patients. However, patients can maintain urinary function for several months to several years after initiating dialysis, though the duration varies according to the cause of the ESRD, comorbidities, dialysis modality, and medication history. While RRF is clinically relevant for various reasons, its integral role in long-term survival of patients is of the greatest importance. A previous study on the relationship between the adequacy of dialysis and survival rate in HD patients showed that survival rate in patients with well-preserved RRF was higher than in their counterparts^2^. A recent study also showed that RRF 1 year after initiating dialysis was the most significant predictor of death in new dialysis patients^1^. However, whether factors, including medication, negatively affect RRF after initiating dialysis has not been conclusive^4,8^. Research has generally shown that the risk of a faster RRF decline increases in both diabetics and patients with heart failure^4,8,13^. RRF is better preserved in patients undergoing peritoneal dialysis than in those undergoing HD^13,19,20^, and, in particular, the risk of reduced RRF for peritoneal dialysis patients is smaller when a RAAS blockade is used, according to a meta-analysis^7^. In contrast, the effects of an RAAS blockade on RRF in HD patients are still uncertain. The latest meta-analysis on the effects of RAAS blockade on RRF included only one study on HD, conducted by Kjaergaard *et al*.^15^, out of the six studies included^21^.

Kjaergaard *et al*. published an RCT on an ARB as a RAAS blocker in 2017. In that study, 82 new dialysis patients (less than 1-year HD vintage) were randomly divided into an irbesartan group or placebo group^15^. The irbesartan group showed relatively low UF volume (irbesartan 0.55 L vs. control 1.30 L at baseline, 1.11 L vs. 1.58 L at 12 months); however, the group did not show a greater gain in preserved RRF. This is consistent with the findings of our study, where the ARB usage rate was high. However, the findings of Kjaergaard *et al*. had low power due to a small sample size, as only 56 of the 82 patients were followed up at 12 months^15^. In contrast, the 12-month follow-up in our study included 376 patients for RRF measurements. Although our study failed to demonstrate an independent protective effect of RAAS blockade, it seemed relatively clear from the results of the LMM modeling (Table 3) and the logistic regression for the development of anuria (Table 4) that the large UF volume had a negative effect on preserving RRF. This finding confirms that RRF preservation requires avoiding inter-dialytic weight gain and intra-dialytic hypotension. Furthermore, while diuretic use was two-fold in the irbesartan group compared to the control in Kjaergaard *et al*.’s study^15^, our findings suggest that diuretics are statistically significantly related to a rapid RRF decline (Table 3). It is not clear that HD patients, as well as PD patients^22^, may benefit from the use of large amounts of diuretics for protecting RRF. Therefore, a well-controlled and designed trial on the effect and safety of diuretic usage in incident HD is warranted. Another RCT was performed with new dialysis patients comparing ACEi (enarapril) and a placebo considering 1-year RRF^16^. That study reported that urine volume in the enarapril group was 700 cc, which was more than twice that for the control group (their baselines were not different). That study measured urine volume for RRF as in our study; however, that study used an ACEi and a small sample of 21 patients in each group^16^, unlike our study. Another RCT investigated the effects of an ultrapure dialysis solution on preserving RRF in new dialysis patients, and the multivariate analysis also failed to demonstrate an independent effect of ACEi on RRF^17^.

Ito *et al*., in 2012, conducted another observational study like present study. They followed 181 patients for another year who showed UF volume of 200 mL or higher 1 year after initiating dialysis^14^. In the follow-up of 110 patients in the RAAS group and 81 patients in the non-RAAS group, the multivariate analysis results suggested a protective effect of RAAS in preserving RRF (HR 0.43, P = 0.026)^14^. However, these results should be interpreted cautiously for three reasons. First, the RAAS group was not clearly defined in that study. In contrast, our study provided a clear definition of the RAAS group as those who used RAAS for 1 year after initiating dialysis based on data between at least two time points (e.g., between the study initiation point and the 3-month point). Second, patients without adverse effects are more likely to maintain RAAS in observational research. Third, patients may already have had a heavily reduced RRF because they were prevalent dialysis patients which has long dialysis vintage. Despite these limitations, that study was important in that it demonstrated the potential of RAAS to preserve RRF.

Although preserving RRF is pivotal in patients undergoing HD from the dialysis initiation, it is difficult to measure properly in clinical practice, so the factors affecting RRF are not well understood, especially in incident HD patients. The traditional method is to measure RRF based on a 24-h urine collection^23^. However, there are several limitations to this method, and alternative methods have been attempted^23–25^. During the first 24 h, it is difficult to collect urine properly, and sometimes urine is collected for more than 24 h^25^. Moreover, urea and creatinine levels change depending on the time of urine collection, and patients who are dialyzed three times per week will have different test values depending on the days of urine collection^25^. According to a recent study published by Lee *et al*. using this cohort, the glomerular filtration rate (GFR) using 24-h urine collection and urine volume were independent risk factors for mortality^26^, but urine volume improves the predictive power of mortality. Therefore, in our study, we decided to use urine volume as an indicator of RRF.

The study limitations need to be clarified. Although our prospective study cohort was relatively large, confounding factors remained compared to RCTs in terms of medication effects, such as the duration of taking a RAAS blocker. Moreover, although we used LMM analysis to adjust for known biasing factors, other uncontrolled differences could have influenced RRF. We also acknowledged the limitation that the measurement of RRF by urine volume may be inaccurate and missing values exist in urine volume in the present study.

In conclusion, we did not demonstrate the clinical benefit of RAAS blockade in protecting RRF and the development of anuria, after adjusting for confounding factors. Further studies are warranted to preserve RRF in Korean patients undergoing HD for the first time.

## Materials and Methods

### Data source and study participants

We organized a nationwide-prospective cohort of ESRD patients at 36 teaching and general hospitals of the Clinical Research Center for ESRD (CRC for ESRD, NCT00931970). The database for analyses performed in this study included patients from August 2008 to December 2014 who had newly started dialysis at the time of study enrollment; 935 patients were finally included (≥18 years) (Fig. 1). Our study cohort protocol and processing for registration at the CRC for ESRD in eligible dialysis patients has been checked and announced^27^. Our study was approved by the institutional review board (IRB) at each center, and all investigators conducted this study in accordance with the guidelines of the 2008 Declaration of Helsinki. All patients provided written informed consent. The Seoul National University Hospital IRB approved this study (IRB number H-0905–047–281). The study was approved by the IRBs at each center as follows [The Catholic University of Korea, Bucheon St. Mary’s Hospital; The Catholic University of Korea, Incheon St. Mary’s Hospital; The Catholic University of Korea, Seoul St. Mary’s Hospital; The Catholic University of Korea, St. Mary’s Hospital; The Catholic University of Korea, St. Vincent’s Hospital; The Catholic University of Korea, Uijeongbu St. Mary’s Hospital; Cheju Halla General Hospital; Chonbuk National University Hospital; Chonnam National University Hospital; Chung-Ang University Medical Center; Chungbuk National University Hospital; Chungnam National University Hospital; Dong-A University Medical Center; Ehwa Womens University Medical Center; Fatima Hospital, Daegu; Gachon University Gil Medical Center; Inje University Pusan Paik Hospital; Kyungpook National University Hospital; Kwandong University College of Medicine, Myongji Hospital; National Health Insurance Corporation Ilsan Hospital; National Medical Center; Pusan National University Hospital; Samsung Medical Center, Seoul; Seoul Metropolitan Government, Seoul National University, Boramae Medical Center; Seoul National University Hospital; Seoul National University, Bundang Hospital; Yeungnam University Medical Center; Yonsei University, Severance Hospital; Yonsei University, Gangnam Severance Hospital; Ulsan University Hospital; Wonju Christian Hospital].

### Definition of the RAAS blockade group

Information about medication being taken was taken from medical records and patient interviews at the time of enrollment into the study, 3 months after enrollment, and every 12 months thereafter. The RAAS group assignment was primarily defined if the patient used an ACEi or ARB at enrolment and continued the medications for at least 3 months.

### Clinical variables

The exact methods for extracting medical history records^26–28^ and evaluating cardiac parameters including 2D-echocardiography^28,29^ have been described previously by the overall CRC for the ESRD cohort (http://webdb.crc-esrd.or.kr). In this study, the following clinical parameters were extracted from the web-based medical records at the time of enrollment into the study cohort: age, sex, primary cause of ESRD, history of diabetes mellitus (DM), cardiovascular disease (CVD), body mass index, and smoking. The MCCI, which has been evaluated in patients with ESRD^30^, was determined by reviewing the patients’ medical histories at the time of enrollment. In our study cohort, left ventricle hypertrophy was defined from the ECG using Cornell’s voltage combination criteria^31^, with the 2D echocardiography procedure performed according to the recommendations of the American Society of Echocardiography^32^. The following parameters were measured from the 2D echocardiogram: left atrial dimension (cm), left ventricle end-systolic dimension (cm), left ventricle end-diastolic dimension (cm), left ventricle mass index (g/m^2^), and left ventricle ejection fraction (%).

### Hemodialysis information

Dialysis information was recorded at the time of stabilized dialysis without changing the dialysis prescription between two weeks after the first dialysis initiation. The first dialysis weekday (Monday or Tuesday) information was recorded. Dialysis-related indices, including ultrafiltration volume per session and week, weekly Kt/v, urea reduction ration (URR), pre-dialysis systolic and diastolic blood pressure, post-dialysis systolic and diastolic blood pressure, and minimum systolic and diastolic blood pressure during dialysis were recorded. In this study, intradialytic hypotension (IDH) was provided according to K/DOQI Clinical Practice Guidelines for Cardiovascular Disease in Dialysis Patients published in 2005^33^. In the K/DOQI guideline, a decrease in systolic BP ≥ 20 mm Hg or decreases in mean arterial pressure (MAP) ≥ 10 mm Hg with typical symptom.

### Measurement of residual renal function

In our cohort, RRF was measured by urine volume per 24 h, and the time schedule that we collect urine 24 h is as follows; the method of urine collection for this study cohort was presented previous study^26^. All of the study participants who agreed to the urine collection were trained by the clinical research coordinator (CRC) for collection method. Baseline and at 3 months, then every 12 months thereafter were collected urine study. Window period could be admitted for ±3 months, but the result of at 3 months for the 24-h urine study showed that 1 month to 3 months could be included. The urine was collected during the 44-h interval between HD sessions and divided into periods to calculate the 24-h urine volume.

### Outcomes

The aim of our study was to evaluate RRF using the change in 24-h urine volume over 0, 3, and 12 months between the RAAS and control groups using mixed-effects linear regression, and a risk factor analysis for the development of anuria 1 year after initiating HD.

### Statistical analysis

Continuous and categorical variables were compared between the RAAS and control groups using Student’s *t*-test for continuous variables and the chi-square test for ordinal variables. This was an observational prospective cohort study with three or more measurement points.

In general, repeated measures ANOVA (rmANOVA) and the linear mixed model (LMM) are used to analyze repeatedly measured data. Selection bias and reduced sample sizes might occur because the model’s assumption that the outcome variable should satisfy a normal distribution and only complete data without missing measurement values were analyzed. The advantage of the LMM for unbalance repeated measures and longitudinal data analysis is that we can describe each individual’s variation pattern despite multiple missing data points^34,35^. In addition, in cases where experimental results are repeatedly obtained from a single experimental subject, erroneous results can be obtained from the use of general linear regression analysis, which assumes the independence of individual measurement data without considering random effects. The LMM assumes that there is a correlation among data repeatedly measured within a single individual (i.e., measures within a subject are correlated).

The present study verified differences in repeatedly measured urine volumes (24-h collected urine volume, ml) using the LMM according to the use of the RAAS blockade (continued medications for at least 3 months, no use) and the time effect (0, 3, and 12 months). Possible differences in RAAS use across 0–12 month visits were analyzed according to interactions between the two variables. As for the LMM’s covariance structures, in order for the characteristics of the data to be best reflected, various models (compound symmetry, autoregressive, and unstructured models) regarding variance–covariance matrix structures were compared, the final model was selected through likelihood ratio tests, and unstructured covariance structures were selected.

The outcome consisted of the main effects (or fixed effects) and concerned whether there were changes in urine volume according to time (within subjects, time effects), whether there were changes in UV according to the use of RAAS drugs (between subject effects, RAAS effects), and whether there were differences between the two groups in changes in UV according to time (interactions). Interactions between time and groups are present when time effects differ according to the group administered drugs, and interactions between time and the groups are said to be absent when time effects do not differ by group. Results with p < 0.05 were considered statistically significant. All statistical analyses were performed using the IBM SPSS Statistics for Windows (Version 24.0; IBM Corp., Armonk, NY, USA) and the SAS (version 9.3; SAS Institute, Cary, NC, USA).

## Supplementary information

Supplement Information

## Data Availability

The datasets are available from the corresponding author upon request.
