# Supplementary material for: Renin Angiotensin Aldosterone System Blockades Does Not Protect Residual Renal Function in Patients with Hemodialysis at 1 Year After Dialysis Initiation: A Prospective Observational Cohort Study
Source: Sci Rep. 2019 Dec 2;9:18103. doi: 10.1038/s41598-019-54572-6 (PMC6889305; doi:10.1038/s41598-019-54572-6)

**Renin Angiotensin Aldosterone System Blockades Does Not Protect Residual Renal Function in Patients with Hemodialysis at 1 Year After Dialysis Initiation: A Prospective Observational Cohort Study**

**Authors**

Kyung Don Yoo^1,*^, Clara Tammy Kim^2,*^, Soie Kwon^3^, Jeonghwan Lee^4^, Yun Kyu Oh^4,5^, Shin-Wook Kang^6^, Chul Woo Yang^7^, Yong-Lim Kim^8^, Yon Su Kim^3,5^, Chun Soo Lim^4,5^, Jung Pyo Lee^4,5^

**Affiliations**

^1^Department of Internal Medicine, Ulsan University Hospital, Ulsan, Korea

^2^Institute of Life and Death Studies, Hallym University, Chuncheon, Korea

^3^Department of Internal Medicine, Seoul National University Hospital, Seoul, Korea

^4^Department of Internal Medicine, Seoul National University Boramae Medical Center, Seoul, Korea

^5^Department of Internal Medicine, Seoul National University College of Medicine, Seoul, Korea

^6^Department of Internal Medicine, Yonsei University College of Medicine, Seoul, Korea ^7^Department of Internal Medicine, The Catholic University of Korea College of Medicine, Seoul, Korea

^8^Department of Internal Medicine, Kyungpook National University School of Medicine, Daegu, Korea

**Supplementary Information**

**Table S1.** Baseline characteristics based on RAAS blockade with preserved residual renal function

**Figure S1.** Mixed-effects linear regression analysis between the three group according to the RAAS inhibitor usage in associated with residual renal function after 1year of dialysis initiation

**Figure S2.** Mixed-effects linear regression analysis between the RAAS group and control group according to the different definition of RAAS group in associated with residual renal function after 1year of dialysis initiation

**Figure S3**. Mixed-effects linear regression analysis between the three group according to the RAAS inhibitor usage according to the different definition of RAAS group in associated with residual renal function after 1year of dialysis initiation

**Table S1. Baseline characteristics based on RAAS blockade with preserved residual renal function**^¶^

| Variables^*^ | | Total N = 480 | | |
| --- | --- | --- | --- | --- |
|  |  | Control group  (N = 249) | RAAS group (N = 231) | *P* |
| Age (years old) | | 57.06 ± 14.38 | 56.88 ± 14.03 | 0.89 |
| Sex (male) | | 160 (64.3%) | 151 (65.4%) | 0.80 |
| Primary renal disease | |  |  | 0.79 |
|  | Diabetes | 132 (53.0%) | 129 (55.8%) |  |
|  | Hypertension | 34 (13.7%) | 28 (12.1%) |  |
|  | Glomerulonephritis | 36 (14.5%) | 33 (14.3%) |  |
|  | Cystic kidney disease | 5 (2.0%) | 5 (2.2%) |  |
|  | Unknown | 18 (7.2%) | 21 (9.1%) |  |
|  | Others | 24 (9.6%) | 15 (6.5%) |  |
| History of CVD | | 79 (31.7%) | 75 (32.5%) | 0.86 |
| History of DM | | 143 (57.4%) | 137 (59.3%) | 0.67 |
| Current smoking history (%) | | 28 (11.2%) | 30 (13.0%) | 0.49 |
| SBP (mmHg) | | 141 ± 20 | 144 ± 22 | 0.18 |
| DBP (mmHg) | | 76 ± 13 | 77± 14 | 0.35 |
| BMI (kg/m2) | | 23.09 ± 3.37 | 22.96 ± 3.26 | 0.68 |
| Modified CCI | | 5.35 ± 2.14 | 5.30 ± 2.37 | 0.81 |
| Concurrent-antihypertensive medications (%) | |  |  |  |
| Calcium channel | | 151 (60.6) | 151(65.4) | 0.28 |
| B-blockers | | 140 (56.2) | 124 (53.7) | 0.57 |
| Diuretics | | 138 (55.4) | 122 (52.8) | 0.56 |
| a-Blockers | | 35(14.1) | 30 (13.0) | 0.73 |
| Cardiologic evaluation | |  |  |  |
| LVH on ECG | | 71 (28.5) | 66 (28.6) | 0.98 |
| cTnT | | 0.99 ± 9.36 | 0.79± 6.23 | 0.82 |
| NT pro-BNP | | 12,581 ± 17,066 | 16,003 ± 21,952 | 0.12 |
| Echocardiographic parameter | |  |  |  |
| LAD (cm) | | 4.26 ± 0.75 | 4.27 ± 0.70 | 0.85 |
| LVESD (cm) | | 3.55 ± 0.78 | 3.53 ± 0.71 | 0.75 |
| LVEDD (cm) | | 5.07 ± 0.68 | 5.19 ± 0.70 | 0.08 |
| LVMI (g/m^2^) | | 209 ± 220 | 210 ± 84 | 0.96 |
| Ejection fraction (%) | | 57.84 ± 11.38 | 59.08 ± 11.01 | 0.29 |

CVD, cardiovascular disease; DM, diabetes mellitus; SBP, systolic blood pressure; DBP, diastolic blood pressure; MCCI, modified Charlson comorbidity index; LVH, left ventricular hypertrophy; cTnT, cardiac troponin T; NT pro-BNP, N-terminal pro-B-type natriuretic peptide; RAAS blockade, renin-angiotensin-aldosterone system blockade

*Values are presented as n (%) for categorical variables, mean ± standard deviation for continuous variables.

^¶^Baseline urine volume more than 500ml for 24 hour urine collection

**Figure S1.** Mixed-effects linear regression analysis between the three groups according to the RAAS inhibitor usage in associated with residual renal function after 1year of dialysis initiation. Adjusted variable including age, sex, dialysis duration, diuretics use, ultrafiltration volume per week, systolic BP, modified Charlson comorbidity index. Green line, RAAS group, 0, 3 months taking RAAS inhibitor; Red line, Control group, never taking all of the three months; Blue line, All remaining observations; Inter-group difference P value = 0.794


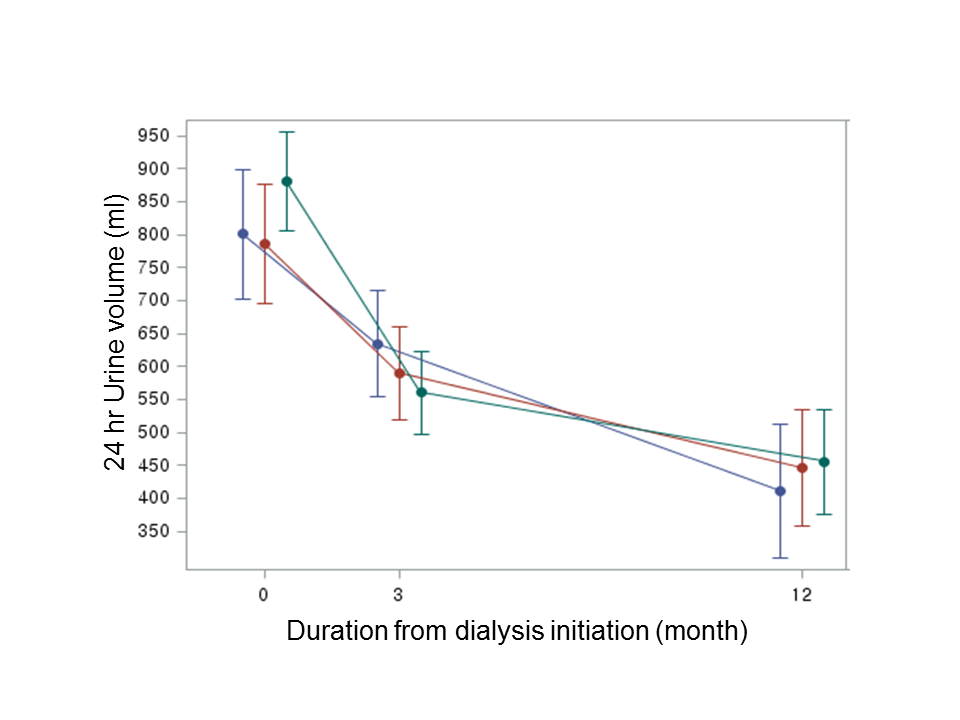


**Figure S2.** Mixed-effects linear regression analysis between the RAAS group and control group according to the different definition of RAAS group in associated with residual renal function after 1year of dialysis initiation. Adjusted variable including age, sex, dialysis duration, diuretics use, ultrafiltration volume per week, systolic BP, modified Charlson comorbidity index. Red line, RAAS group, 0, 3 and 12 months taking RAAS inhibitor; Blue line, Control group, All remaining observations; Inter-group difference P value = 0.165


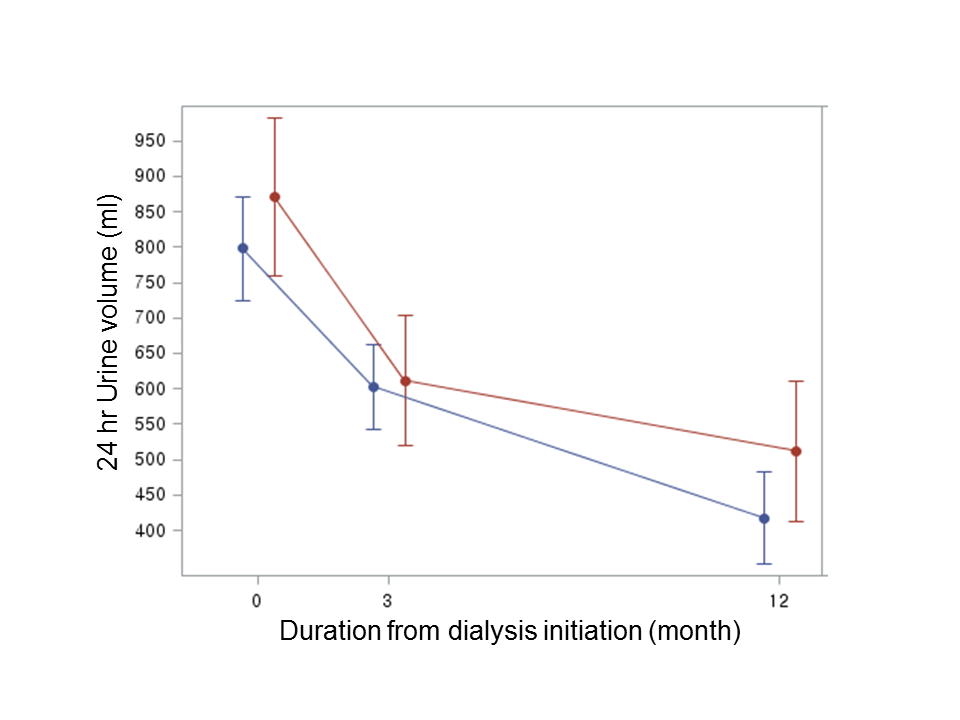


**Figure S3**. Mixed-effects linear regression analysis between the three group according to the RAAS inhibitor usage according to the different definition of RAAS group in associated with residual renal function after 1year of dialysis initiation. Adjusted variable including age, sex, dialysis duration, diuretics use, ultrafiltration volume per week, systolic BP, modified Charlson comorbidity index. Green line, RAAS group, 0, 3, and 12 months group; Red line, Control group, never taking all of the RAAS inhibitor at 0, 3, 12 months; Blue line, All remaining observations, Inter-group difference P value = 0.326


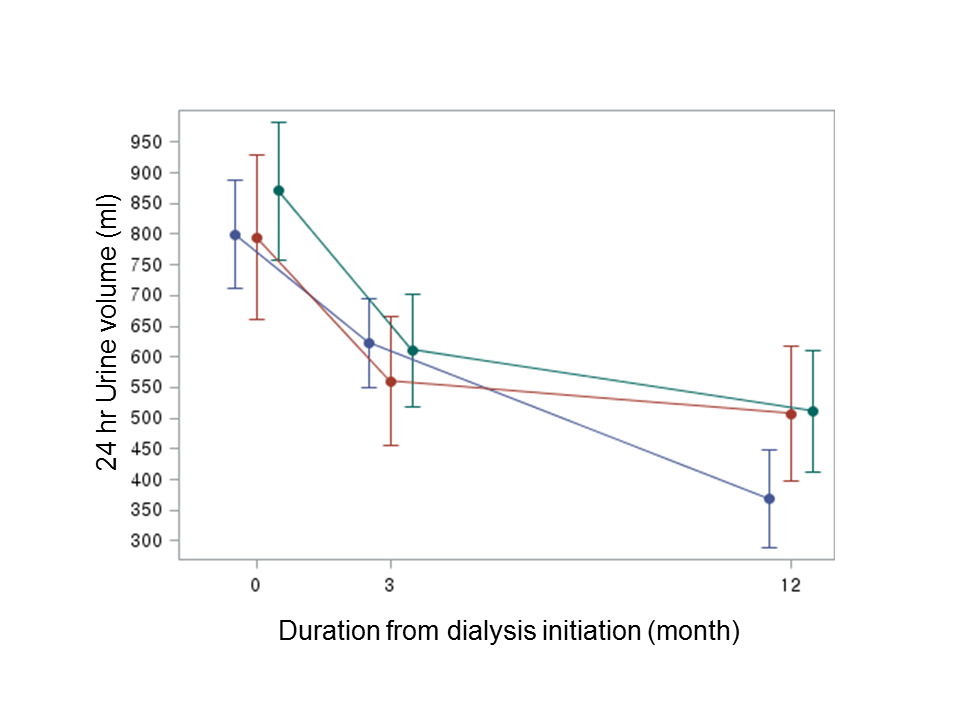

Supplement: Supplementary file 1 — Supplement Information [file 41598_2019_54572_MOESM1_ESM.docx]
